# Substitution of Thr572 to Ala in mouse c-Myb attenuates progression of early erythroid differentiation

**DOI:** 10.1038/s41598-020-71267-5

**Published:** 2020-09-01

**Authors:** Kyoko Kitagawa, Chiharu Uchida, Ryo Horiguchi, Tatsuya Ohhata, Satoshi Sakai, Hiroyuki Niida, Shuhei Yasumoto, Yukino Handa, Moena Suzuki, Masako Hashimoto, Toshiyasu Tazawa, Yuta Yokochi, Mayumi Tsuji, Masatoshi Kitagawa

**Affiliations:** 1 Department of Molecular Biology, Hamamatsu University School of Medicine, 1-20-1 Handayama, Higashi-ku, Hamamatsu, Shizuoka 431-3192 Japan; 2 Advanced Research Facilities & Services, Preeminent Medical Photonics Education & Research Center, Hamamatsu University School of Medicine, Hamamatsu, 431-3192 Japan; 3 Department of Virology and Parasitology, Hamamatsu University School of Medicine, Hamamatsu, 431-3192 Japan; 4 Department of Environmental Health, University of Occupational and Environmental Health, Kitakyushu, 807-8555 Japan; 5 Laboratory Animal Facilities & Services, Preeminent Medical Photonics Education & Research Center, Hamamatsu University School of Medicine, Hamamatsu, 431-3192 Japan

**Keywords:** Ubiquitylation, Haematopoietic stem cells

## Abstract

The expression level of transcription factor c-Myb oscillates during hematopoiesis. Fbw7 promotes ubiquitin-mediated degradation of c-Myb, which is dependent on phosphorylation of Thr572. To investigate the physiological relevance of Fbw7-mediated c-Myb degradation, we generated mutant mice carrying c-Myb-T572A (TA). Homozygous mutant (TA/TA) mice exhibited a reduction in the number of peripheral red blood cells and diminished erythroblasts in bone marrow, presumably as a result of failure during erythroblast differentiation. We found that c-Myb high-expressing cells converged in the Lin^−^CD71^+^ fraction, and the expression of c-Myb was higher in TA/TA mice than in wild-type mice. Moreover, TA/TA mice had an increased proportion of the CD71^+^ subset in Lin^−^ cells. The c-Myb level in the Lin^−^CD71^+^ subset showed three peaks, and the individual c-Myb level was positively correlated with that of c-Kit, a marker of undifferentiated cells. Ultimately, the proportion of c-Myb^hi^ subgroup was significantly increased in TA/TA mice compared with wild-type mice. These results indicate that a delay in reduction of c-Myb protein during an early stage of erythroid differentiation creates its obstacle in TA/TA mice. In this study, we showed the T572-dependent downregulation of c-Myb protein is required for proper differentiation in early-stage erythroblasts, suggesting the in vivo significance of Fbw7-mediated c-Myb degradation.

## Introduction

Ablation of c-Myb expression by gene targeting indicates that c-Myb, a member of the hematopoietic transcription factor family, is essential for fetal liver hematopoiesis and erythroid development^1,2^. c-Myb is consistently expressed at high levels in immature progenitors of all hematopoietic lineages and is associated with the regulation of proliferation, differentiation, and survival of progenitors^3^. Levels of c-Myb decrease during terminal differentiation to mature blood cells^4^; tetracycline-regulated expression of c-Myb in c-Myb^−/−^ embryonic stem (ES) cells prevents the terminal differentiation of erythrocytes and megakaryocytes^5^. These observations prove that appropriate levels of c-Myb protein are strictly defined in distinct stages of differentiation of the hematopoietic cell lineage to maintain normal proliferation and differentiation. Consistent with this role, several c-Myb target genes linked to proliferation and differentiation, such as c-Myc and c-Kit, have been identified in myeloid cells^2^.

The cellular abundance of c-Myb is controlled not only by transcription but also by post-transcriptional mechanisms. c-Myb is highly expressed in immature erythroid progenitors with the level of expression decreasing during maturation. It is suggested that the decrease in c-Myb is due to a block in transcription elongation^6,7^. It is thought that c-Myb undergoes post-translational modifications, including phosphorylation and ubiquitylation, resulting in the c-Myb protein having a short half-life^8^. Accordingly, a Skp1/Cul/F-box protein (SCF)-type E3 ubiquitin ligase, SCF-Fbw7/Fbxw7, targets c-Myb for ubiquitin-dependent proteolysis via the proteasome^9,10^.

The F-box protein Fbw7 is a substrate-binding module for the SCF-type E3 ubiquitin ligase, and Fbw7 regulates the abundance of several transcription factors such as c-Myb, Notch1^11,12^, c-Myc^13,14^, GATA2^15^, and GATA3^16^, which regulate differentiation of hematopoietic cells. Hence, Fbw7 contributes to the quiescence of hematopoietic stem cells (HSC) and the regulation of differentiation and proliferation of progenitor cells; loss of Fbw7, therefore, might lead to complex phenotypes resulting from dysregulated stability of its multiple target proteins.

Although conditional *Fbw7*-knockout (KO) mice develop thymic lymphomas, defects of HSC, or failure of erythrocyte maturation, the function of Fbw7 in chronic myeloid leukemia is tumour-promoting^17,18^. Depletion of Fbw7 reduces levels of hemoglobin and platelets^19^. These findings indicate that Fbw7 plays different roles in diverse situations.

**Figure 1:**
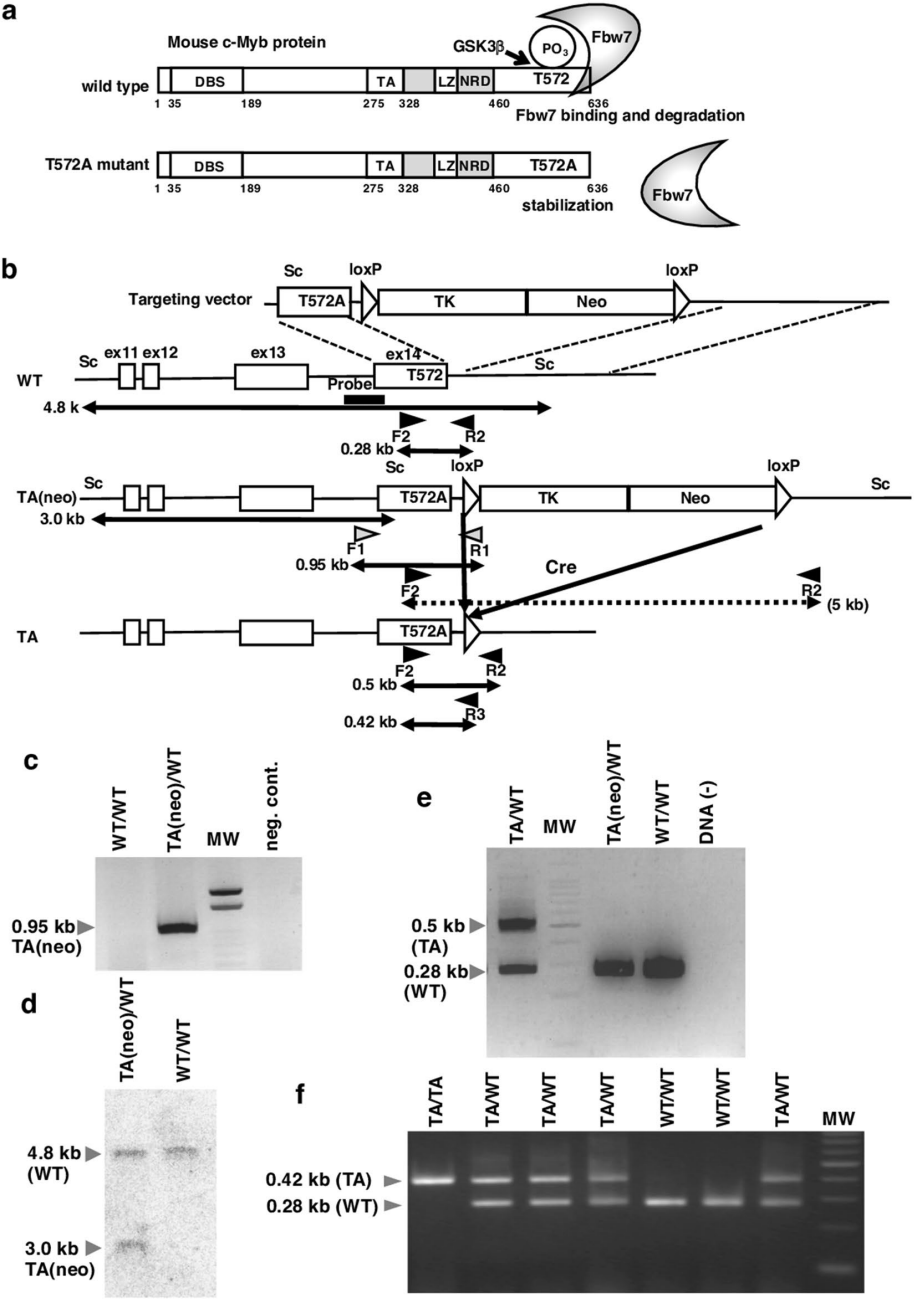
Construction of c-Myb-T572A knock-in mice. (**a**) The diagram of Fbw7-mediated regulation of T572-dependent mouse c-Myb. (**b**–**f**) Generation of mice carrying c-Myb-T572A. (**b**) The targeting construct encoding the murine *c-Myb* gene modified to express c-Myb-T572A (targeting vector) and schematic representations of the wild-type mouse *c-Myb* allele (WT); the recombinant *c-Myb* allele, which resulted from T572A amino acid exchange with a loxP-thymidine kinase (TK)-neomycin resistant (Neo) cassette [TA(neo)]; and the *c-Myb*-T572A knock-in allele after removal of the TK-Neo cassette by Cre recombinase (TA). Exons and loxP sites are shown as open boxes and triangles, respectively. The expected sizes of bands in a PCR analysis with primer set 1 (F1 and R1), primer set 2 (F2 and R2), and primer set 3 (F2 and R3) and a Southern blot analysis with probe [for SacI (Sc) fragments] are indicated. (**c**,**d**) Screening of homologous recombinant embryonic stem (ES) clone by PCR analysis (**c**) and Southern blot analysis (**d**). (**c**) Genomic DNA from ES clones were subjected to PCR with the primers F1 and R1. The position of the amplified fragment (0.95 kb) is indicated in (**b**). (**d**) Genomic DNA was digested with *Sac*I and subjected to hybridization with probe. The wild-type and homologous recombinant alleles gave rise to hybridizing fragments of 4.8 and 3.0 kb, respectively. (**e**) Confirmation of a floxed ES clone. Genomic DNA from ES clones was analyzed by PCR with the primers F2 and R2. The positions of amplified fragments corresponding to wild-type (0.28 kb), homologous recombinant (5 kb) and knock-in (0.5 kb) alleles are indicated in (**b**). (**f**) Genotyping of *c-Myb* DNA extracted from F_7_ mouse ears. The position of primers (F2, R2, and R3) used for the genotyping is indicated in (**b**).

In a previous study, we reported that depletion of Fbw7 resulted in accumulation of c-Myb protein and decreased the abundance of *γ-globin* transcripts, which is repressed by c-Myb in human myeloid leukemia K562 cells^10^. This finding suggests that endogenous Fbw7 plays a role in regulating c-Myb as a pivotal E3 ligase for c-Myb degradation in myeloid leukemia cells. Fbw7 targets not only c-Myb but also Notch, c-Myc, GATA2, and GATA3, all of which are hematopoietic factors. Therefore, we cannot distinguish the specific hematopoietic failure responsible for deregulation of c-Myb in *Fbw7* KO mice. Fbw7 often recognises substrates by phosphorylation status on consensus phosphopeptide binding motif for Cdc4, termed “Cdc4 phosphodegron”. In our previous study, we clarified that GSK3-mediated phosphorylation of Thr572 in mouse c-Myb was required for the binding of Fbw7 and its degradation by Fbw7^10^ (Fig. 1a). To further investigate the role of Fbw7-mediated c-Myb degradation in control of hematopoietic cell lineage development in a physiologically relevant manner, we generated mutant mice that did not undergo Fbw7-mediated c-Myb degradation. c-Myb in which Thr572 was replaced by Ala could not bind to Fbw7 and thus was resistant to Fbw7-mediated proteasomal degradation^10^. Therefore, using a classical knock-in strategy in the current study, we generated mice expressing the T572A mutant of c-Myb. Consequently, by following the decline in c-Myb protein level during hematopoietic differentiation, we identified the starting point of the decrease. Here, we showed that diminution of c-Myb in early differentiation, which was disturbed in T572A knock-in mice, is required for the precise progression of differentiation.

## Results

### Generation of mice carrying c-Myb-T572A mutant

Our previous study indicated that Fbw7 recognizes and binds to mouse c-Myb in a phosphorylation of T572 dependent manner and that the c-Myb-T572A mutant protein is more stable than WT c-Myb in HeLa cells, despite the presence of Fbw7^10^ (Fig. 1a). To confirm whether c-Myb-T572A mutant has transcriptional activity equivalent to that of WT c-Myb, we conducted a reporter assay using pHSE-Luc, which contains a heat shock element (HSE) sequence. c-Myb protein binds to heat shock factor 3 (HSF3) and enhances activation of pHSE-Luc^20^. The exogenous expression level and relative activity of c-Myb T572A mutant in HeLa cells were almost equivalent to those of c-Myb WT, suggesting that the c-Myb mutant (with Thr572 to Ala substitution) retained its transcriptional potency (Supplementary Fig. S1). Therefore, generation of mutant mice carrying c-Myb-T572A is useful for analyzing the physiological significance of Fbw7-mediated degradation of c-Myb in vivo.

To generate the mutant mice that escape binding of Fbw7 and subsequent degradation, we designed a targeting construct to replace the WT *c-Myb* allele with an allele in which the codon for Thr572 was changed to an Ala codon (Fig. 1a,b). The targeting vector was introduced into mouse ES cells by electroporation, and transfectants were selected based on their ability to grow in the presence of G418. Homologous recombination events were confirmed by PCR (Fig. 1c) and Southern blot analysis (Fig. 1d). The *loxP*-flanked thymidine kinase-neomycin resistance cassette was then deleted by infection of an adenovirus vector encoding Cre recombinase to the selected ES cells, and infectants were selected based on their ability to grow in the presence of gancyclovir using PCR screening (Fig. 1e). The targeted ES cells were cloned and injected into C57BL/6 blastocysts, and chimeric male mice that transmitted the mutant allele in the germ line were obtained. Heterozygotes were bred to produce c-Myb-T572A homozygous mice. Genotyping was performed by PCR using mixed primers F2, R2, and R3, in which F2-R2 amplified the WT allele (0.28 kb) and F2-R3 specifically amplified the TA allele (0.42 kb) (Fig. 1b,f). Although F2-R2 amplified both the WT (0.28 kb) and TA (0.5 kb) alleles in ES cells, this primer set amplified the TA allele in mice at very low efficiency, probably because of the different conditions of DNA preparation.

### c-Myb-T572A homozygous knock-in mice exhibit putative anemia

**Figure 2:**
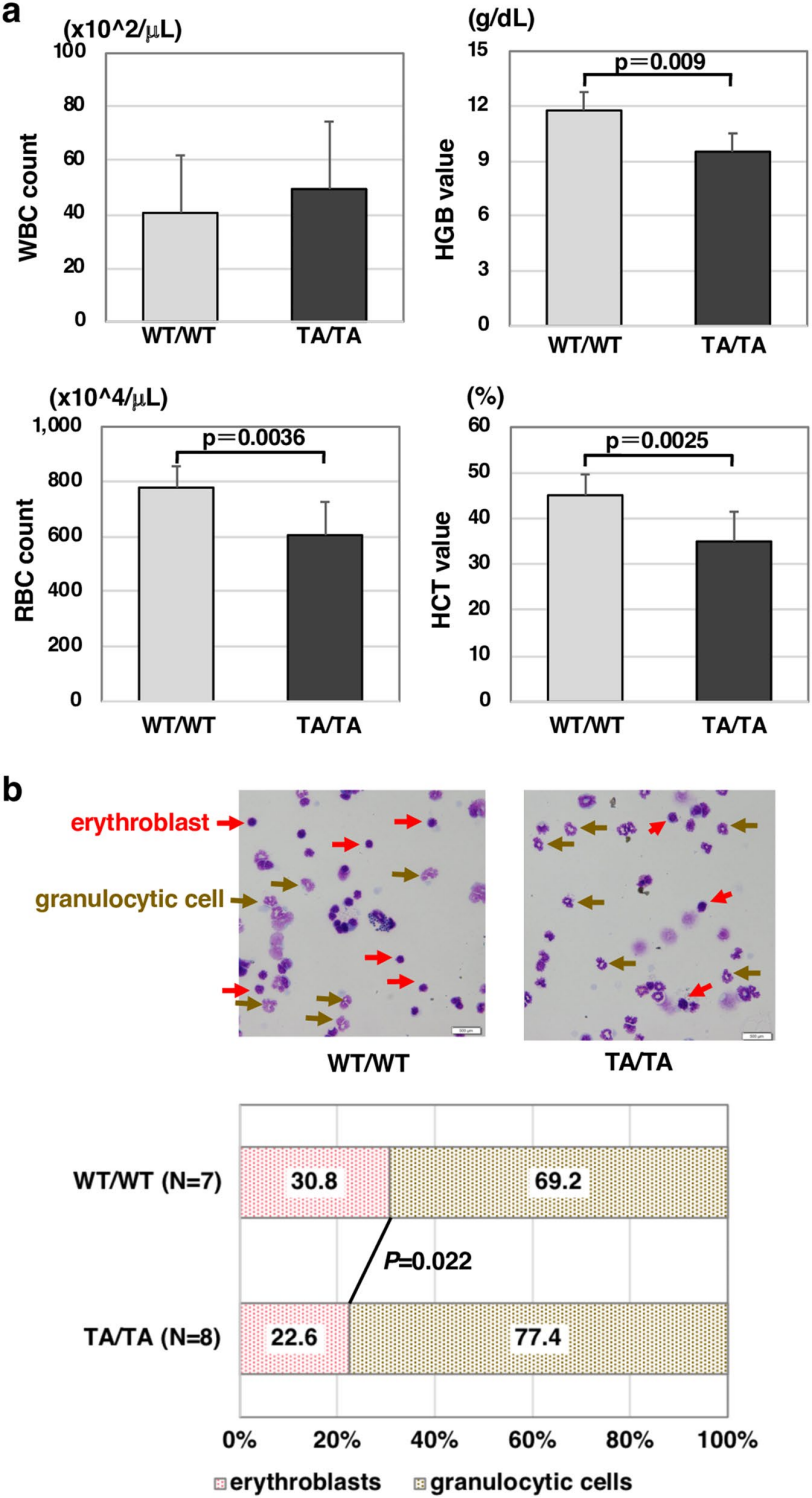
Occurrence of anemia in c-Myb-T572A homozygous knock-in mice. (**a**) Peripheral blood cell count and erythrocyte-related analytical parameters of the c-Myb-T572A homozygous knock-in (TA/TA; black bars, *n* = 7) and control (WT/WT; gray bars, *n* = 9) mice. Results are shown as means ± SD. WBC, white blood cell; RBC, red blood cell; HGB, hemoglobin; HCT, hematocrit. (**b**) The morphology of granulocytic cells and erythroblasts in the bone marrow of TA/TA (*n* = 7) and WT/WT (*n* = 8) mice. Hematopoietic cells in bone marrow of TA/TA or WT/WT mice were subjected to May-Grünwald Giemsa staining. Red arrows, erythroblasts; brown arrows, granulocytic cells; original magnification ×40.

First, we analyzed the peripheral blood cells in c-Myb-T572A homozygous knock-in (TA/TA) and c-Myb wild-type (WT/WT) mice. The number of red blood cells, but not white blood cells, was significantly reduced in TA/TA mice (Fig. 2a). Moreover, levels of hemoglobin and hematocrit were significantly lower in TA/TA than in WT/WT mice (Fig. 2a). To assess immature blood cells, BM progenitor cells were obtained from femurs of WT/WT and TA/TA mice, and thin-layer cell was prepared by cytocentrifuge and stained with May-Grünwald Giemsa (Fig. 2b, upper). The ratio of erythroblasts to myelocytes in BM progenitor cells was significantly decreased in TA/TA mice compared with WT/WT control mice (Fig. 2b, bottom). These results suggest two possibilities: (1) enhancement of myelocyte progression, or (2) repression of erythrocyte differentiation, proliferation, or both, in TA/TA mice. Considering the peripheral blood cell counts, the latter may be more likely.

### Fluctuation of c-Myb level during differentiation of hematopoietic cells

**Figure 3:**
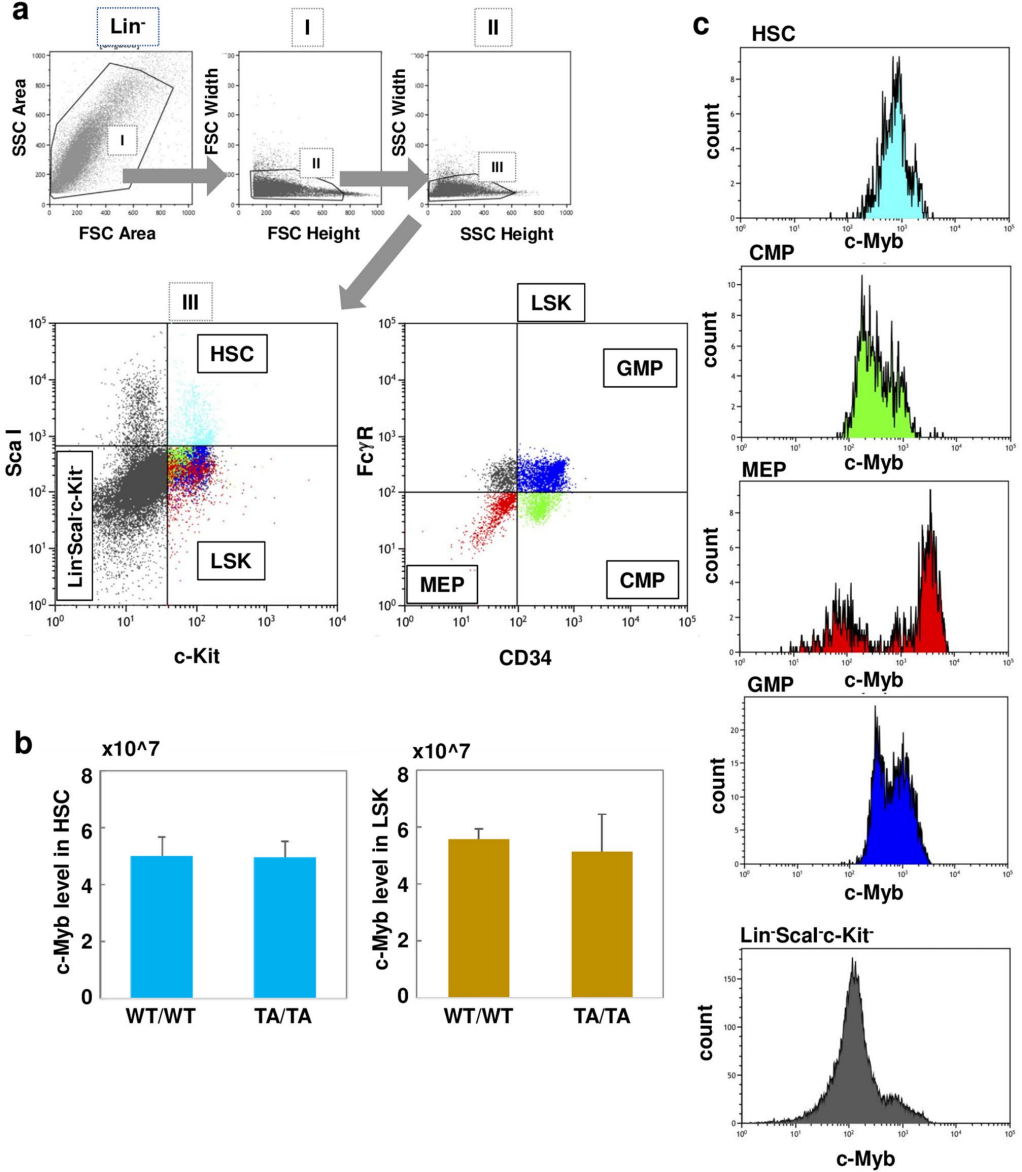
Fluctuation of c-Myb level during differentiation of hematopoietic cells. (**a**) Gating strategy for Lin^−^ScaI^+^c-Kit^+^ hematopoietic stem cells (HSC), Lin^−^ScaI^−^c-Kit^+^ (LSK), and Lin^−^ScaI^−^c-Kit^−^ progenitor fractions from the bone marrow mononuclear cell fraction that was negative for interleukin (IL)-7 receptor and lineage-affiliated antigens (Lin^−^). The LSK fraction was subdivided into FcγR^lo^CD34^+^, FcγR^hi^CD34^+^, and FcγR^lo^CD34^−^ populations, representing common myeloid progenitors (CMP), granulocyte/macrophage progenitors (GMP), and megakaryocyte/erythroid progenitors (MEP), respectively. FSC, forward scatter; SSC, side scatter. (**b**) Relative c-Myb expression level in HSC or LSK subsets was compared between WT/WT (*n* = 5) and TA/TA mice (*n* = 6). Results are shown as means ± SD. (**c**) Relative c-Myb expression levels for each population were identified by histogram analysis.

To evaluate these possibilities in detail, we determined the state of hematopoietic progenitors of TA/TA mice. First, we prepared the BM mononuclear cell fraction that was negative for lineage-affiliated antigens and IL-7 receptor. This fraction was subdivided into ScaI^+^c-Kit^+^ (Lin^−^ScaI^+^c-Kit^+^, a typical surface marker pattern of HSC), ScaI^−^c-Kit^+^ (Lin^−^ScaI^−^c-Kit^+^, LSK), and ScaI^−^c-Kit^−^ (Lin^−^ScaI^−^c-Kit^−^, which belongs to a late-lineage progenitor fraction), according to a FACS analysis scheme (Fig. 3a). We confirmed proliferation potential of HSC and LSK subsets by colony forming cells (CFC) assay (Supplementary Table S1). The WT/WT and TA/TA mice did not differ significantly in populations of HSC and LSK subsets; furthermore, the protein level of c-Myb in WT/WT and TA/TA mice was equivalent in HSC and LSK subsets (Fig. 3b).

The LSK cells were then fractionated into CMP, GMP, and MEP populations according to the expression patterns of FcγR and CD34, as previously reported^21^; CMP, GMP, and MEP were defined as FcγR^lo^CD34^+^, FcγR^hi^CD34^+^, and FcγR^lo^CD34^−^ populations, respectively (Fig. 3a). It has been reported that expression of CD71, an effective marker for erythroid precursors, is detected not only in MEP (about 82%) but also in CMP (about 32%) in human BM^22^. Interestingly, c-Myb expression in MEP showed multiple peaks (Fig. 3c), presumably reflecting the + /− CD71 population. Therefore, we next investigated c-Myb level during differentiation of erythroid progenitors using anti-CD71 antibody, as described below.

### Delayed decrease in c-Myb level and compromised differentiation of erythroid progenitors in c-Myb-T572A mice

**Figure 4:**
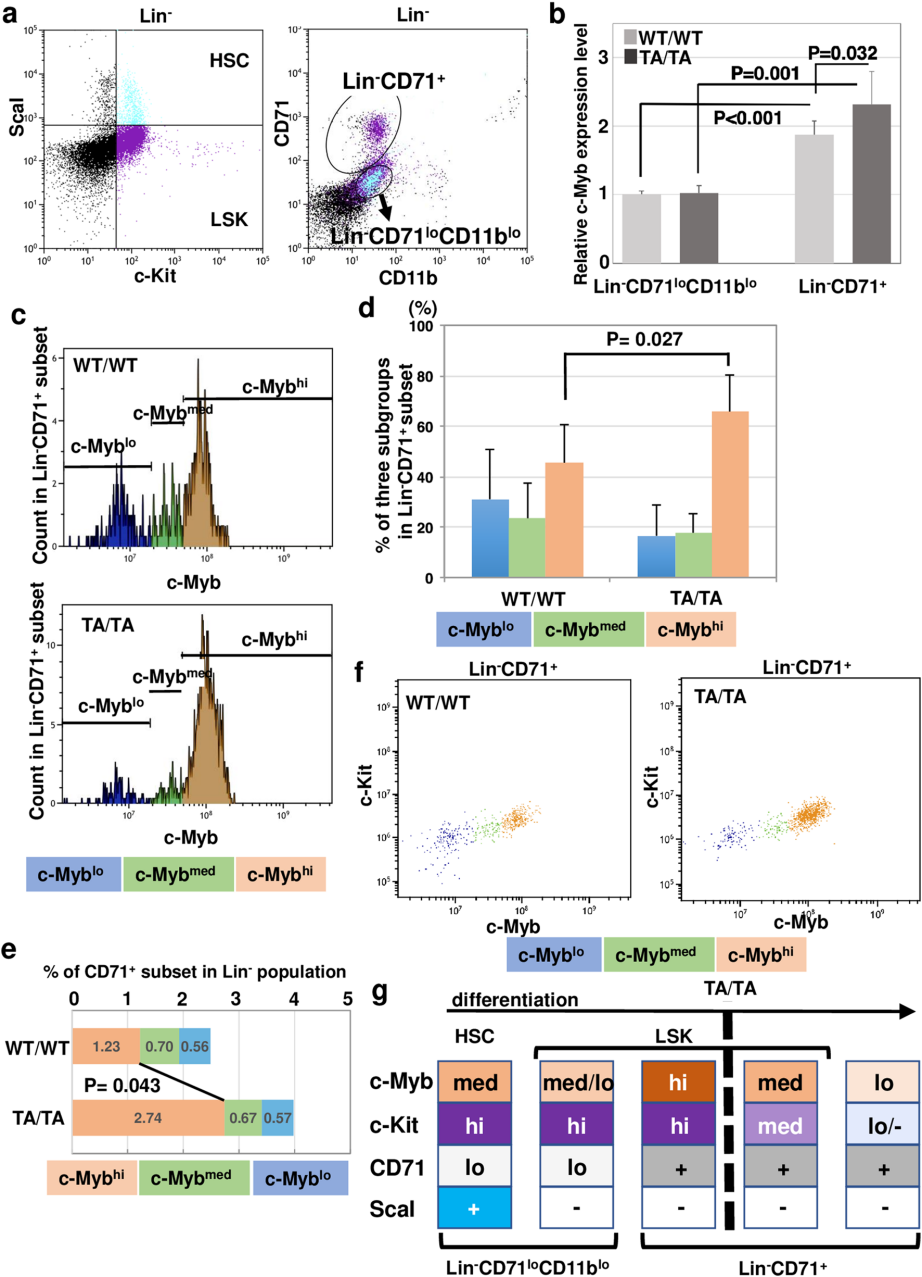
Diminished decrease in c-Myb according to differentiation of erythroid progenitor and attenuation of its development in TA/TA mice. (**a**) In Lin^−^ cells, LSK and HSC subsets were detected with anti-ScaI and c-Kit antibodies, and CD71^lo^CD11b^lo^ and CD71^+^ subsets were identified using anti-CD71 and CD11b antibodies simultaneously. (**b**) Relative expression levels of c-Myb in CD71^lo^CD11b^lo^ and CD71^+^ subsets from Lin^−^ cells in WT/WT mice (*n* = 6) or TA/TA mice (*n* = 5) were determined. Results are shown as means ± SD. *P*-values were estimated using Student’s *t*-test. (**c**) The Lin^−^CD71^+^ subset was further divided into three subgroups (c-Myb^lo^, c-Myb^med^, and c-Myb^hi^) according to c-Myb expression. Representative histograms of WT/WT and TA/TA mice were shown. (**d**) Existence of the three subgroups in the Lin^-^CD71^+^ subset derived from histogram data (**c**) and compared between WT/WT (*n* = 6) and TA/TA mice (*n* = 5). *P*-values were estimated using Student’s *t*-test. (**e**) The proportions of CD71^+^ fraction in Lin^−^ cells, which consists of three subgroups with different c-Myb expression levels, were compared between WT/WT mice (*n* = 6) and TA/TA mice (*n* = 5). *P*-values were estimated using Student’s *t*-test. (**f**) The correlation between c-Myb expression and c-Kit in Lin^−^CD71^+^ subset was ascertained. (**g**) The schema proposed in this study is shown, indicating fluctuations in c-Myb and 3 lineage markers (c-Kit, CD71, and ScaI) during differentiation of the Lin^−^ erythroid lineage of wild-type mice. The broken line indicates the stage at which progression is disturbed in the Lin^−^ erythroid lineage of TA/TA mice.

Lin^−^ progenitors were subdivided into LSK and HSC subsets using anti-ScaI and c-Kit antibodies (Fig. 4a, left). They were simultaneously separated using anti-CD71 and anti-CD11b antibodies to clarify the state of erythroid progenitors (Fig. 4a, right). Lin^−^CD71^lo^CD11b^lo^ subgroups included a part of the LSK (violet dots) and HSC (blue dots) (Fig. 4a, right). The c-Myb levels in Lin^−^CD71^+^ progenitor cells were significantly higher than those of Lin^−^CD71^lo^CD11b^lo^ progenitor cells in both wild-type (*P* < 0.001) and TA/TA mice (*P* = 0.001) (Fig. 4b). Because the expression of CD71 and CD11b in the progenitor cells represents the erythroid lineage and the granulocyte or macrophage lineage, respectively, we considered that LSK cells that did not express CD71 or CD11b belonged to an earlier differentiation stage than the Lin^−^CD71^+^ subset. Consequently, we postulated that an increase in c-Myb level in the Lin^−^CD71^+^ subset is required for progression from Lin^−^CD71^lo^CD11b^lo^ progenitors. Furthermore, the c-Myb level in Lin^−^CD71^+^ progenitor cells was significantly higher in TA/TA mice than in WT/WT mice (*P* = 0.032) (Fig. 4b). These results indicate that c-Myb levels in Lin^−^CD71^+^ progenitor cells, which were increased during the progression of differentiation from Lin^−^CD71^lo^CD11b^lo^, were significantly enhanced in TA/TA mice compared with wild-type mice.

Focusing on the Lin^−^CD71^+^ subset, we noted that the expression of c-Myb showed three peaks (Fig. 4c). We then evaluated the ratios of the three subgroups. In the Lin^−^CD71^+^ fraction, the proportion of the c-Myb^hi^ subgroup was significantly increased in TA/TA mice compared with WT/WT mice (*P* = 0.027); in contrast, c-Myb^lo^ and c-Myb^med^ subgroups were decreased in TA/TA mice (Fig. 4d). Moreover, the proportion of the CD71^+^ fraction of the c-Myb^hi^ subgroup in the Lin^−^ fraction was significantly increased in TA/TA mice compared with wild-type mice (*P* = 0.043), although the proportions of c-Myb^lo^ and c-Myb^med^ subgroups were equal between TA/TA mice and wild-type mice (Fig. 4e). These results suggest that elevated c-Myb in Lin^−^CD71^+^ progenitor cells affects differentiation in the erythroid lineage in TA/TA mice.

c-Myb is reported to play a role in expression of c-Kit in hematopoietic cells^23,24^. Consistently, expression of c-Myb in Lin^−^CD71^+^ cells was positively correlated with c-Kit level in both mice genotypes (Fig. 4f, Supplementary Fig. S2). Because c-Kit is a marker of undifferentiated hematopoietic cells, we assumed that the Lin^−^CD71^+^c-Myb^hi^ subgroup belonged to an early stage of the erythroblast lineage. It also appeared that an increase in erythroblast progenitor cells in the early stage, as observed in TA/TA mice, resulted in a failure of differential progression.

The fractionated c-Kit^+^ or c-Kit^−^ subset in Lin^−^C71^+^ was used to evaluate the mRNA level of *c-Myb* in each differential stage. We showed that the c-Kit^+^ subset had much higher *c-Myb* mRNA than the c-Kit^−^ subset and that *c-Myb* mRNA in TA/TA mice was about half that in WT/WT mice in both subsets (Supplementary Fig. S3a). It implies that repressed transcription takes place to compensate for translational or post translational effects, which might cause increased c-Myb protein level in CD71^+^Lin^−^ progenitor cells of TA/TA mice. Taken together, these results suggest that a transcriptional decrease of *c-Myb* participates in reduction of the c-Myb protein level during differentiation of erythroblast progenitor. Moreover, the results indicate that the increase in c-Myb level in Lin^−^CD71^+^ cells of TA mice, as shown in Fig. 4b, was caused by a failure of post-transcriptional regulation such as ubiquitin-mediated degradation of c-Myb protein. We noted that the c-Kit^+^ subset had significantly higher *Fbw7* mRNA than the c-Kit^−^ subset (Supplementary Fig. S3b). It makes sense that Fbw7 promotes downregulation of c-Myb in Lin^−^c-Kit^+^CD71^+^ subset to induce differentiation to the Lin^−^c-Kit^−^CD71^+^ subset.

The delay in reduction of c-Myb might have resulted in increase of the proportion of the CD71^+^ fraction of the c-Myb^hi^ subgroup in the Lin^−^subset, leading to an obstacle to differentiation in TA/TA mice. Thus, our results suggest that a decrease in c-Myb protein level followed by a decrease in c-Kit level is required for normal differentiation of Lin^−^CD71^+^ erythroblasts and this process was hampered in TA/TA mice, which retained a high level of c-Myb protein (Fig. 4g).

## Discussion

During erythropoiesis, c-Myb expression is restricted in the early developmental stages, which include HSC status and LSK^25^. In the later stage, c-Myb is rapidly downregulated, which is required to permit differentiation^26^. Related to these fluctuations, higher (deregulated) expression of c-Myb is often detected in human acute myelogenous leukemia^27^. In this study, we detected a fluctuation in c-Myb level during erythroid differentiation. During the early stage of progenitor differentiation, c-Myb was increased in part of the MEP subset relative to HSC (Fig. 3c). By immunoblot analysis with mouse tissue lysates using clone 1–1 anti c-Myb antibodies, we also observed that c-Myb expression was abundant in the thymus but was not detectable in the BM, which is mainly composed of Lin^+^ cells (Supplementary Fig. S4a). Moreover, we detected equal levels of endogenous mouse c-Myb protein in the thymus of WT/WT and TA/TA mice (Supplementary Fig. S4a). However, the sorted Lin^−^c-Kit^+^ subset, which also belongs to BM-derived cells (about 10% of the population), showed higher expression of c-Myb than thymocytes, which we confirmed by immunoblot assay (Supplementary Fig. S4b). These results prove that c-Myb levels fluctuate during differential progression in BM. Expression of c-Myb was highest in the ScaI^−^c-Kit^+^CD71^+^ subgroup, subsequently diminishing in concert with c-Kit; however, this fluctuation was disrupted in TA/TA mice (Fig. 4d–g, Supplementary Fig. S2). The expression of Fbw7 in the LSK fraction was shown by Takeishi et al.^28^. We previously indicated Fbw7-dependent degradation of c-Myb in a K562 cell line that was established from human leukemia^10^, moreover, we observed that mRNA expression of *Fbw7* was markedly higher in the Lin^−^c-Kit^+^CD71^+^ subset than in the Lin^−^c-Kit^−^CD71^+^ subset (Supplementary Fig. S3b). These findings are consistent with our hypothesis that Fbw7 plays a role in downregulating c-Myb levels in the Lin^−^c-Kit^+^CD71^+^ subset during transition from the Lin^−^c-Kit^+^CD71^+^ to the Lin^−^c-Kit^−^CD71^+^ stage.

Fbw7 is a critical antioncogenic factor because of its suppression of oncogenic substrates such as cyclin E, c-Myc, Mcl-1, mTOR, Jun, Notch, and AURKA^29^. Loss of Fbw7 in hematopoietic cells or T cells of conditional knockout mice is sufficient to cause T-cell acute lymphoblastic leukaemia (T-ALL) or thymic lymphoma, in which increased levels of Notch1 and c-Myc are seen^19,30^. Loss of Fbw7 also results in Myc accumulation in development of chronic myelogenous leukemia (CML) and ALL^31^. Moreover, multiple activating gene mutations, including c-Myb, take place in T-ALL^32^. In addition, c-Myb is required for BCR/ABL-dependent myeloid leukemia onset^33^. Hematopoietic progenitor cells and breast cancer tissue often overexpress c-Myb^34,35^. In light of these reports, we expected that TA/TA mice would be prone to developing tumors. During this study, we found some dysplasia formations from accumulation of lymphocytes in liver or kidney of TA/TA mice. Nevertheless, we could not conclude that TA/TA mice have any tumorigenic character because the frequency was low. Furthermore, we did not perceive an effect on survival caused by cancers on TA/TA mice during breeding for 1 year. In consideration of these results, the disturbance of c-Myb in TA/TA mice may be inadequate for carcinogenesis. The disorder of other factors including p300 and C/EBPβ could cooperate with accumulation of c-Myb to promote carcinogenesis. Further experiments are needed to clarify this. Practically, not only c-Myb, but also other transcription factors such as C/EBPβ and c-Myc are noted to be therapeutic targets in the acute myeloid leukemia process^36^. Although TA/TA mice do not seem to be cancer prone, at least in the present study, our experimental results suggest that Fbw7-mediated c-Myb degradation is required for the quantitative regulation of c-Myb in early erythroid progenitors at the stage when c-Kit disappears, and failure of c-Myb downregulation causes attenuated erythroid differentiation and induces anemia in TA/TA mice.

Genome-wide studies have identified numerous genes as targets of c-Myb^37,38^; therefore, the importance of c-Myb in development, cell survival, proliferation, and homeostasis is unequivocal^7^. However, it is not yet clear what type of transcriptional regulation is lost as a critical event resulting from c-Myb reduction during progenitor differentiation. Both c-Kit and c-Myb are the markers of immature cells and these molecules are expressed in HSC; in addition, the decrease in both factors was synchronized (Fig. 4f, Supplementary Fig. S2). Because it is known that c-Myb functions as a transcriptional activator of c-Kit expression and continuous c-Kit expression interferes with cell differentiation, failure of c-Myb degradation may cause defect of erythrocyte progression in TA/TA mice.

Finally, in TA/TA mice, progression of the Lin^−^CD71^+^ fraction seemed to be attenuated because of aberrant fluctuations in c-Myb level. We presume that this disruption in differentiation results in an insufficient supply of mature erythrocytes.

## Materials and methods

### Cell culture

HeLa cells were maintained in Dulbecco’s modified Eagle medium supplemented with 10% fetal bovine serum, penicillin (100 units/mL), and streptomycin (100 μg/mL).

### Plasmids

The expression plasmid of c-Myb wild-type (WT) was previously described^10^, and we constructed the T572A mutant type using standard recombinant DNA techniques. The luciferase reporter plasmid (pHSE-Luc) was purchased from Clontech Laboratories (Mountain View, CA, USA).

### Antibodies

Pacific Blue anti-c-Kit (2B8), phycoerythrin (PE)/Cy7 anti-Sca-1 (E13-161.7), and PE or allophycocyanin (APC)/Cy7 anti-Fcγ R III/II (93) were purchased from Biolegend (Tokyo, Japan). Fluorescein isothiocyanate (FITC) anti-CD34 (RAM34) was purchased from eBioscience (San Diego, CA, USA). Biotin Mouse Lineage Depletion Cocktail, biotin anti-CD127 (B12-1), PE anti-CD71 (C2), and FITC anti-CD11b (M1/70) were purchased from BD Biosciences (Tokyo, Japan). Alexa Fluor 647 anti-c-Myb (phosphor S11) (EP769Y) was purchased from Abcam (Tokyo, Japan). Anti-Myb mouse monoclonal antibody (1-1) was purchased from Millipore (Tokyo, Japan). The availability of clone EP769Y toward mouse c-Myb was confirmed by immunoblot assay using mouse thymocyte lysate (Supplementary Fig. S4b), and the equivalent sensitivity of that toward WT and T572A c-Myb was confirmed by immunoblot assay using His-tagged exogenous proteins and the antibodies against His tag or c-Myb, the clone EP769Y or the clone 1-1 (Supplementary Fig. S5).

### Luciferase assay

HeLa cells were transfected with the luciferase reporter plasmid, β-galactosidase (β-gal) expression plasmid, and c-Myb expression plasmid or empty vector using Fugene6 (Promega, Madison, WI, USA). The total amount of transfected DNA was the same in each experiment. Cells were lysed at 48 h after transfection and assayed for luciferase and β-gal activities. Luciferase activity was normalized to that of β-gal.

### Generation of c-Myb^T572A/T572A^ knock-in mice

The codon for Thr572 in exon 14 was mutated to an Ala codon to create the *c-Myb*-T572A allele. The targeting vector was constructed by insertion of both a PGK-TK-poly(A) cassette and a PGK-neo cassette between two *loxP* sequences in intron 14 of *c-Myb* (Fig. 1b). The maintenance, transfection, and selection of mouse ES cells were performed as described previously^39^. G418-resistant clones were screened for homologous recombinants by subjecting cell lysates to PCR with a forward primer F1 (5′-ACAGCACTTCACCGTCTTAGTG-3′) and a cassette-specific reverse primer R1 (5′-AAAGGGCCTCGTGATACGCCTA-3′) (Fig. 1b), the annealing sites and expected PCR product size of which are shown in Fig. 1b. The results of PCR screening were confirmed by Southern blot analysis; DNA prepared from PCR-positive ES clones was digested with SacI, fractionated by electrophoresis, transferred to a nylon membrane (Biodyne B; Pall Corp., Tokyo, Japan), and then hybridized with a 0.7-kb probe. The expected sizes of hybridizing fragments for WT and recombinant alleles were 4.8 and 3.0 kb, respectively (Fig. 1b,d). The *loxP*-flanked PGK-TK-poly(A) cassette and PGK-neo cassette were deleted in transfected ES cells by infection with an adenovirus encoding Cre recombinase. Gancyclovir-resistant clones were screened for T572A mutant (TA) clones by PCR with a forward primer F2 (5′-CTTACGAGCTCTGTTTTAATGGCACCTGTATC-3′) and a reverse primer R2 (5′-CTGAACAAGAATTTCCAGTGTCTCAC-3′) (Fig. 1e), the annealing sites of which are shown in Fig. 1b. The expected sizes of the amplified fragments for WT and knock-in alleles were 0.28 and 0.5 kb, respectively (Fig. 1b,e). The mutant ES cells were microinjected into C57BL/6 blastocysts, and the resulting male chimeras were mated with female C57BL/6 mice. Heterozygous offspring were intercrossed to produce homozygous mutant animals. The knock-in mice were at 7 generations of backcross onto the C57BL/6 background. All mice were treated according to the protocols approved by the Hamamatsu University School of Medicine Animal Care Committees at the Laboratory Animal Facilities & Services. All offspring were tested for the presence of the TA or WT *c-Myb* alleles by PCR with primers F2, R2, and R3 (5′-TAGTGAACCTCTTCGAGGGACCT-3′) (Fig. 1f). We used both male and female mice between 8 and 15 weeks of age as the sources of peripheral blood or bone marrow (BM) cells for analysis. The mice were generated according to protocols approved by the Hamamatsu University School of Medicine Animal Care Committees at the Center Animal Care facility (approval number; 2017076, approval date; 2017.10.17).

### Blood counts

Seven WT/WT mice and nine TA/TA mice were bled into tubes containing EDTA-2K, and blood counts were obtained at Falco Holdings (Kyoto, Japan). The mean and standard deviation of counts were calculated.

### BM cell observation

The morphology of BM cells was confirmed by Cytospin preparation and May-Grünwald Giemsa staining. Thin-layer cell preparation was performed by using the Cytospin 4 cytocentrifuge (Thermo Fisher Scientific, Tokyo, Japan) according to the manufacturer’s protocol. The collected cells were subsequently stained with May-Grünwald Giemsa (Merck, Darmstadt, Germany) and the images were captured by using a BX51 microscope at 40× magnification (Olympus, Tokyo, Japan).

### Fluorescence-activated cell sorting (FACS) analysis

Mouse BM mononuclear cells from the femur were enriched by Ficoll density gradient centrifugation using Ficoll-Paque PLUS (GE Healthcare, Tokyo, Japan). Subsequently, lineage cells and common lymphoid progenitor populations were magnetically depleted with biotin-labelled antibodies against CD127; the Biotin Mouse Lineage Depletion Cocktail contained biotinylated monoclonal antibodies to mouse CD3e, CD11b, CD45R/B220, Ly-6G, Ly-6C, and TER-119 and streptavidin-conjugated magnetic particles (BD Biosciences, Tokyo, Japan). The enriched Lin^−^CD127^−^ (Lin^−^) population was fixed and permeabilized by using the Foxp3 staining kit (eBioscience), which is applicable to staining of nuclear proteins before intracellular c-Myb staining or cell-surface marker staining. The Lin^−^CD127^−^ population was subdivided into common myeloid progenitors (CMP), granulocyte/macrophage progenitors (GMP), and megakaryocyte/erythroid progenitors (MEP) as Lin^−^ScaI^−^c-Kit^+^CD34^+^FcγR^lo^, Lin^−^ScaI^−^c-Kit^+^CD34^+^FcγR^hi^, and Lin^−^ScaI^−^c-Kit^+^CD34^−^FcγR^lo^ populations, respectively, as previously reported^21^. The Lin^−^CD127^−^ population was also stained with anti-CD71 and anti-CD11b in addition to anti-ScaI and anti-c-Kit to recognize erythroid and myelocyte progenitors, which were enriched within the Lin^−^CD71^+^ and Lin^−^CD11b^+^ populations, respectively. All stained cells were scored by MoFlo Astrios (Beckman Coulter, Tokyo, Japan) and reanalyzed using Kaluza Analysis software (Beckman Coulter).

### Immunoblot analysis

Tissue lysates were extracted with RIPA buffer, and hematopoietic cells and thymocytes were lysed in lysis buffer (0.3% Triton X-100, 300 mM NaCl, 50 mM Tris–HCl, pH 7.5). Forty micrograms of tissue and cell lysates of a fixed number were separated by SDS-PAGE and transferred from the gel onto a PVDF membrane (Millipore, Tokyo, Japan), followed by immunoblotting. Proteins were visualized using an enhanced chemiluminescence system (Bio-Rad Laboratories, Hercules, CA, USA).

### Gene expression analysis

Total RNA was isolated from the fractionated c-Kit^+^ or c-Kit^−^ subset in Lin^−^CD71^+^ progenitor cells using RNAiso Blood (Takara) and subjected to reverse transcription with oligo dT primer and SuperScript Reverse Transcriptase IV (Invitrogen). The resulting complementary DNA was subjected to quantitative real time PCR using the StepOnePlus system (Applied Biosystems) and a TaqMan probe (Mm005017141_m1, Applied Biosystems), or Clarity digital PCR system (JN Medsys, Singapore) using SYBR mix (Thunderbird SYBR qPCR mix, TOYOBO, Osaka, Japan) and the Fbw7-specific primers that we described previously^16^. Transcripts were normalized to the number of fractionated cells.

### Statistical analysis

Data are presented as mean ± SD. Comparisons between two groups were analysed by Student’s *t*-test, where *P* < 0.05 was considered statistically significant.

## Supplementary information

Supplementary Information 1.
