# Supplementary material for: Substitution of Thr572 to Ala in mouse c-Myb attenuates progression of early erythroid differentiation
Source: Sci Rep. 2020 Sep 1;10:14381. doi: 10.1038/s41598-020-71267-5 (PMC7463259; doi:10.1038/s41598-020-71267-5)

Substitution of Thr572 to Ala in mouse c-Myb attenuates progression of early erythroid differentiation

Kyoko Kitagawa, Chiharu Uchida, Ryo Horiguchi, Tatsuya Ohhata, Satoshi Sakai, Hiroyuki Niida, Shuhei Yasumoto, Yukino Handa, Moena Suzuki, Masako Hashimoto, Toshiyasu Tazawa, Yuta Yokochi, Mayumi Tsuji and Masatoshi Kitagawa

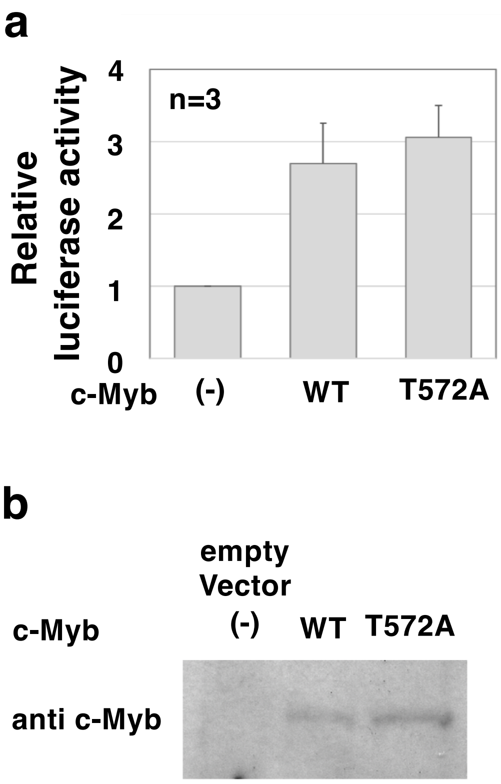

Supplementary Figure S1. Confirmation of transcriptional activity of T572A c-Myb. HeLa cells were transfected with the luciferase reporter: β-gal expression plasmid, wild-type or T572A mutant c-Myb expression plasmid or empty vector. Cells were lysed to measure luciferase and β-gal activities. Activity of luciferase was normalized to that of β-gal and results presented as the relative ratio of the sample transfected with empty vector (**a**). The data shows the mean ± SD of three independent experiments. The expression levels of c-Myb were evaluated by immunoblotting using 1.5 % of total cell lysate and anti c-Myb antibody (**b**).

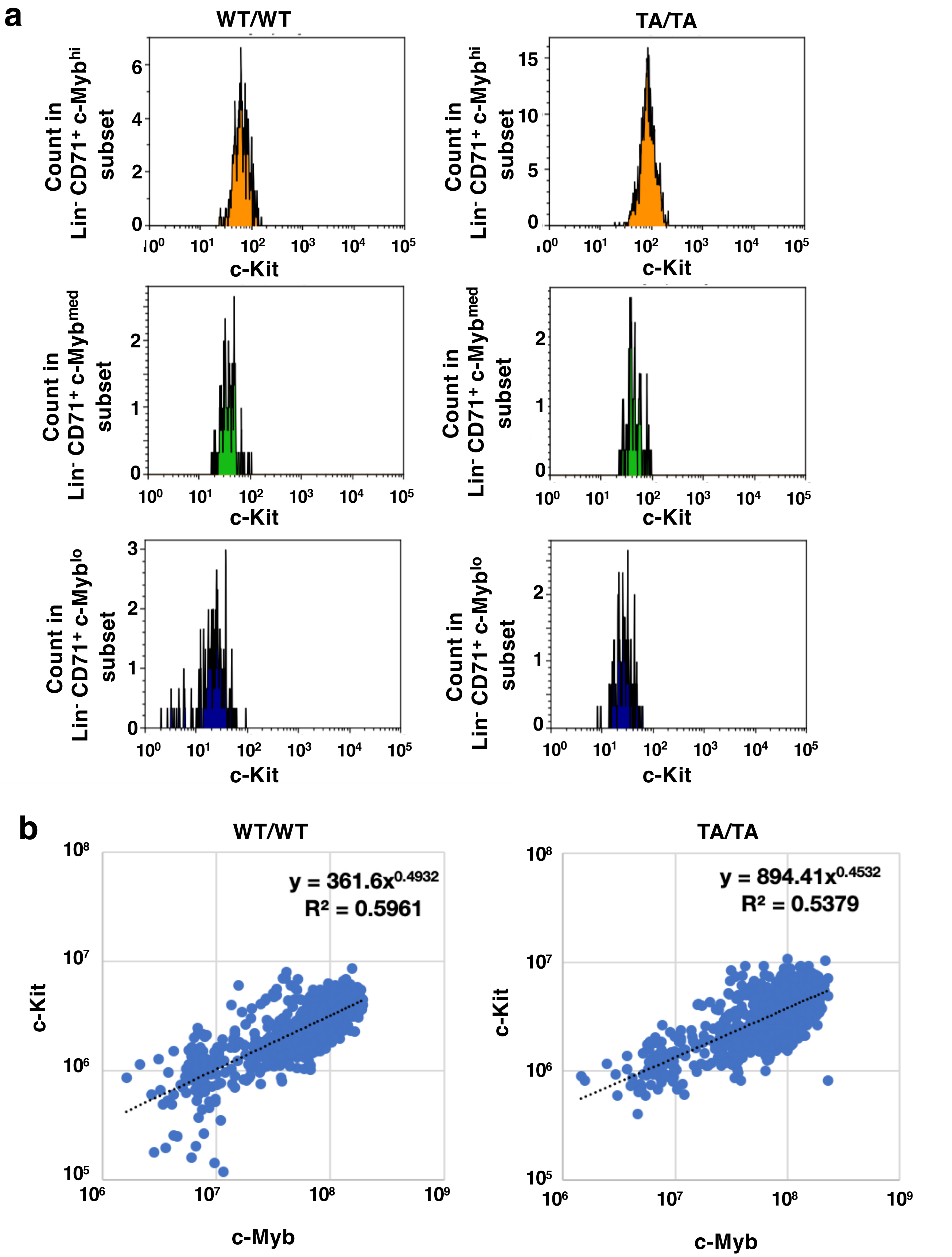

Supplementary Figure S2. Validation of positive correlation between c-Myb level and c-Kit level in Lin$^-$CD71$^+$ subset. (**a**) Lin$^-$CD71$^+$ subset was subdivided into three subgroups (c-Myb^{lo}, c-Myb^{med}, and c-Myb^{hi}) according to c-Myb expression. Histogram shows the representative expression of c-Kit in each subgroup of WT/WT and TA/TA mice. (**b**) The relationship between expression level of c-Myb and that of c-Kit was evaluated with nonlinear regression analysis.

a

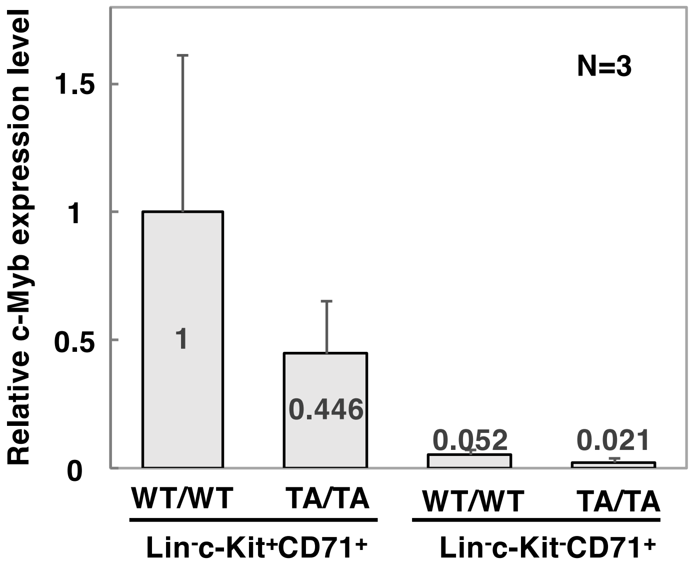

b

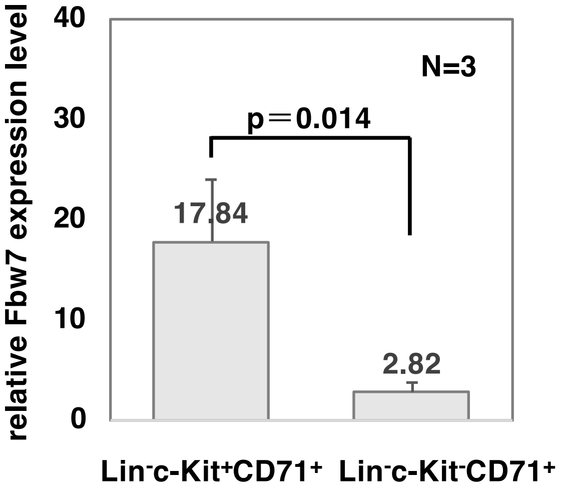

Supplementary Figure S3. Evaluation of the transcriptional levels of c-Myb and Fbw7 at a differential stage of Lin^-CD71^+ subset. Total RNA was extracted from the fractionated c-Kit^+ or c-Kit^- subset in Lin^-C71^+ to prepare cDNA. The amounts of transcripts were corrected by the number of sorted cells. Data are means ± SD from three mice of each genotype. *P*-values were estimated using Student's *t*-test. (**a**) Expression of c-Myb was evaluated using StepOnePlus real time PCR system (Applied Biosystems, Foster City, CA, USA) and a TaqMan probe (Mm005017141_m1, Applied Biosystems). (**b**) The transcription of Fbw7 was evaluated by Clarity digital PCR system (JNmedsys, Singapore) using SYBR mix (Thunderbird SYBR qPCR mix, TOYOBO, Osaka, Japan) and the Fbw7-specific primers that we described previously[16].

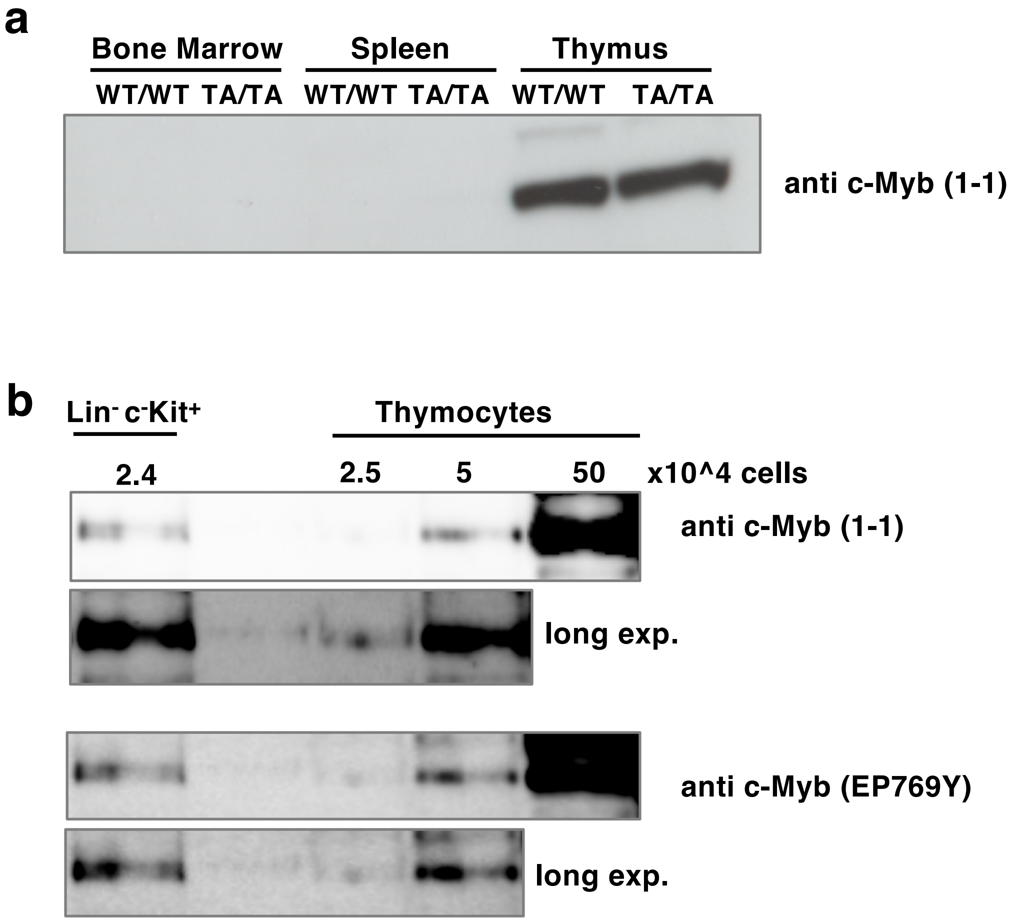

Supplementary Figure S4. Detection of endogenous c-Myb protein in mouse tissue and cells by immunoblotting. Mouse tissue and cell lysates were prepared with lysis buffer containing protease inhibitors. (**a**) Forty micrograms of tissue lysate and (**b**) the sorted Lin$^-$c-Kit$^+$ subset in bone marrow and thymocytes were subjected to immunoblot analysis with 2 clones of antibodies against c-Myb to compare expression level of c-Myb.

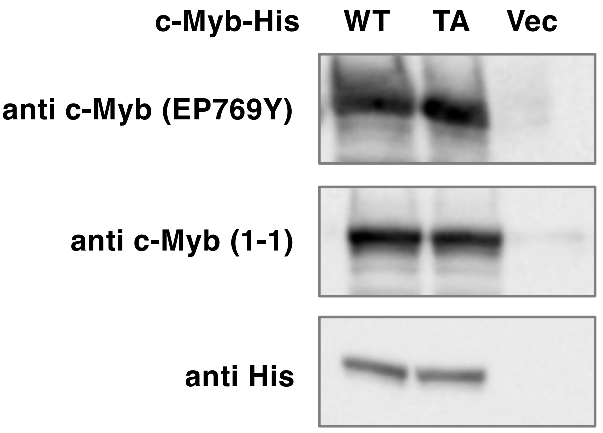

Supplementary Figure S5. Detection of exogenous c-Myb protein by immunoblot analysis. His-tagged wild-type (WT) and T572A c-Myb proteins expressed in HEK293T cells were subjected to immunoblot detection. Sensitivity of antibodies against c-Myb, clone EP769Y (used in FACS analysis in this study; Abcam) or clone 1-1 (validated for western blotting; Millipore), were compared with antibody against His tag.

		CFC	
**subset**	**plating**	**number**	**%**
**HSC**	**400**	**109**	**27.3**
**LSK**	**1600**	**131**	**8.2**

Supplementary Table S1. Confirmation of proliferation potential of HSC and progenitor cells. For the colony forming cell (CFC) assay, HSC and LSK progenitors were directly sorted into MethoCult (M3434, StemCell Technologies, Vancouver, BC, Canada), using anti-ScaI and anti-c-Kit antibodies from $Lin^{-}CD127^{-}$ cells. A total of 400 and 1600 cells were divided into 4 and 8 wells of a 12-well plate for HSC and LSK, respectively. Colonies were counted 7 days after plating.

uncropped data **Figure 1**

c

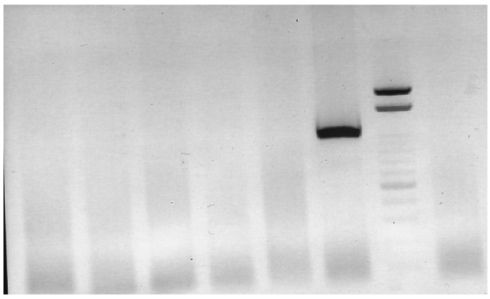

d

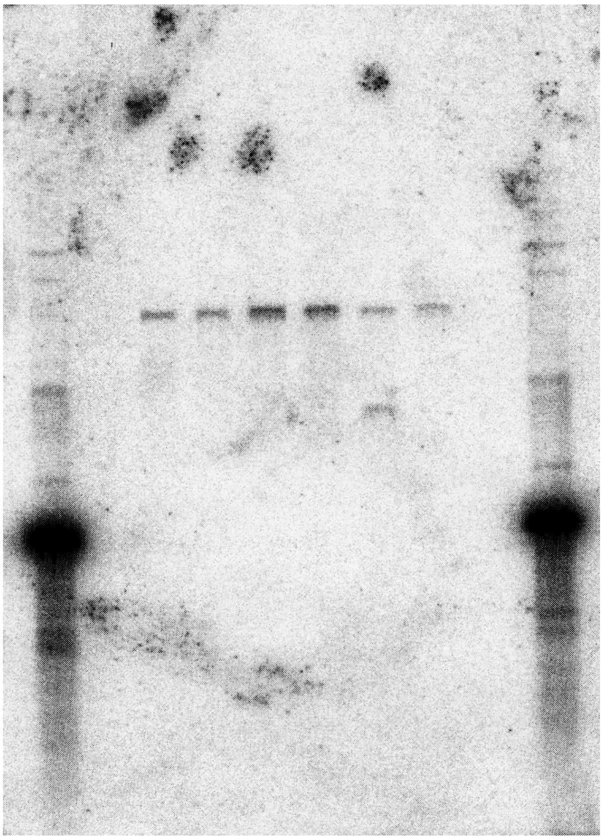

e

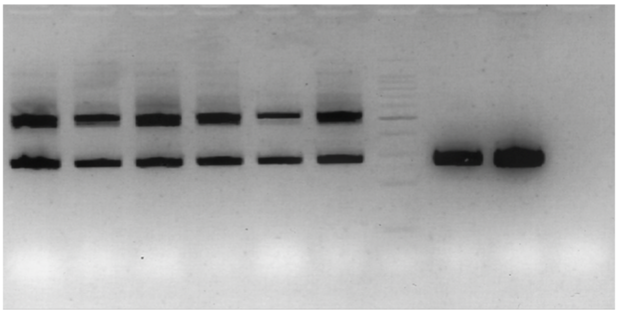

uncropped data

Supplementary Figure S1

a

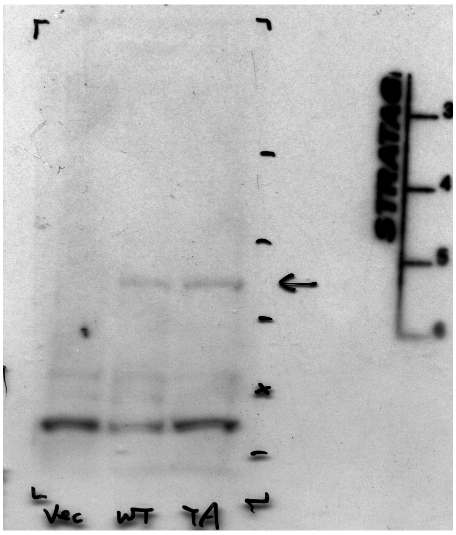

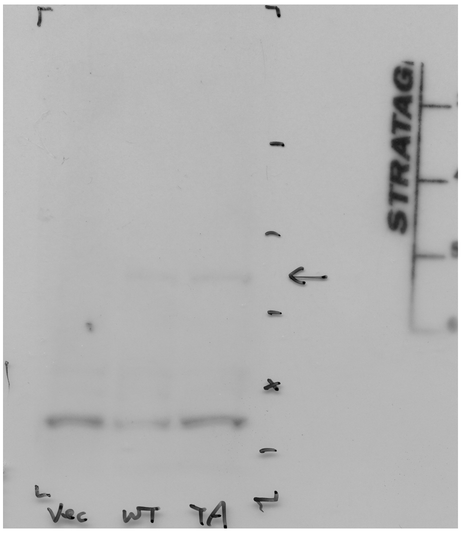

original

uncropped data

Supplementary Figure S4

a

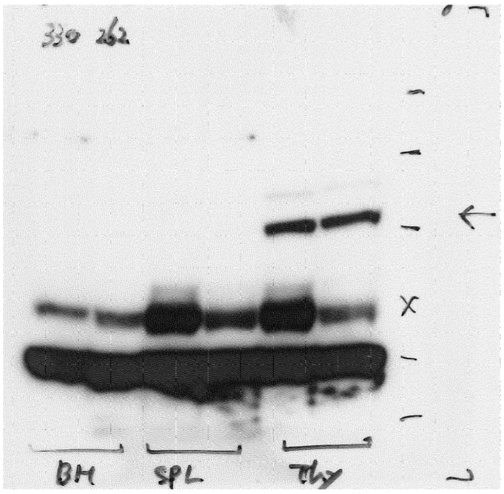

b

anti c-Myb (1-1)

anti c-Myb (EP769Y)

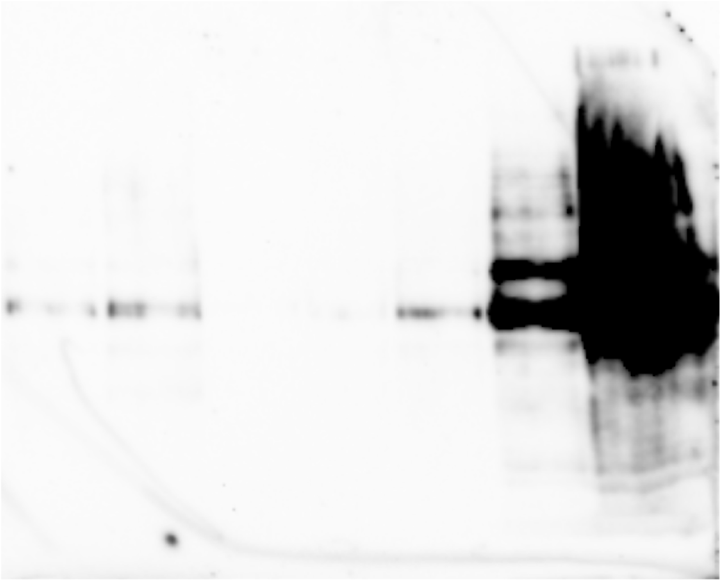

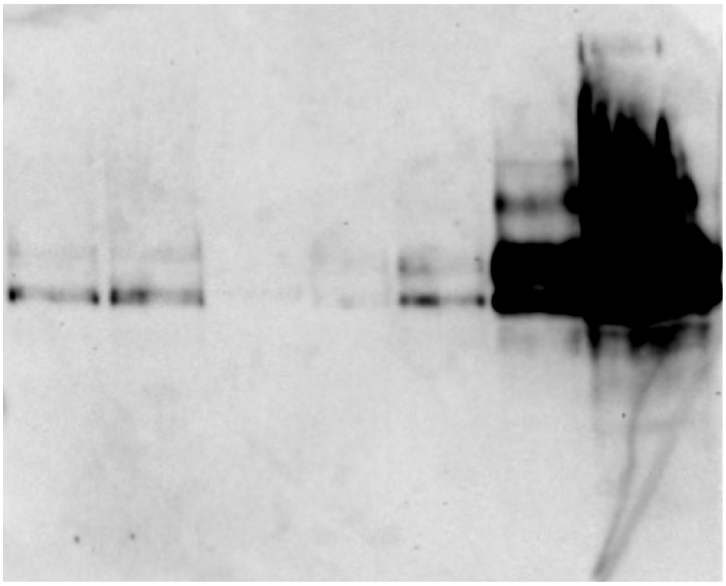

long exp.

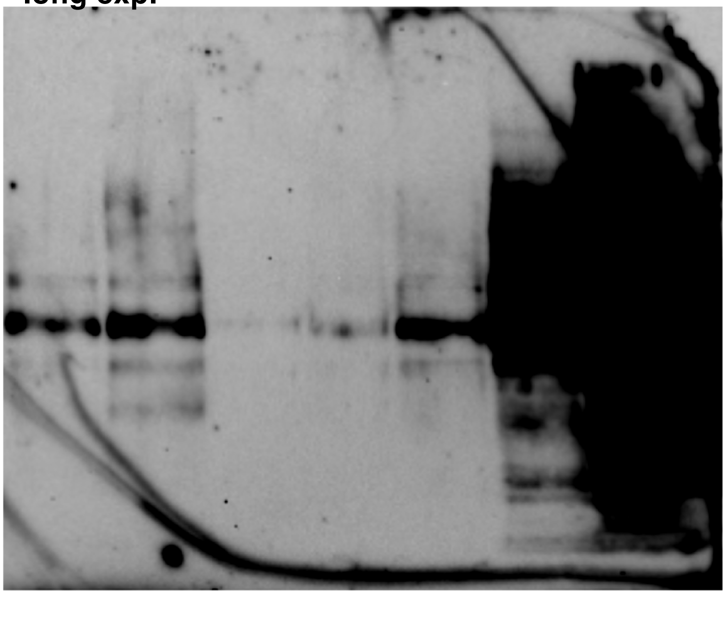

long exp.

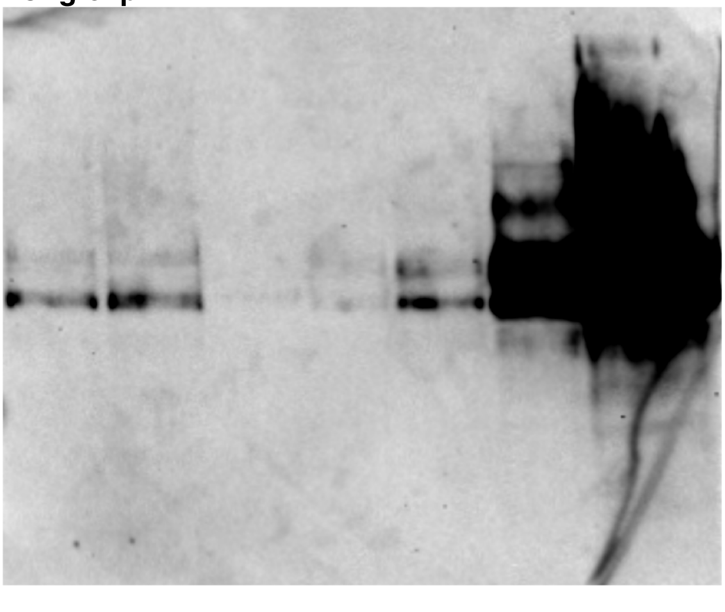

uncropped data

Supplementary Figure S5

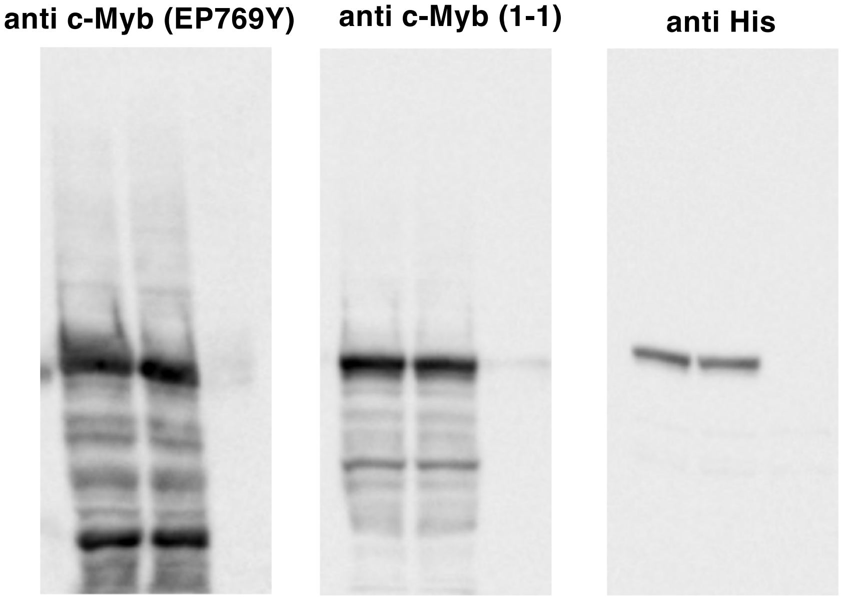

Supplement: Supplementary file 1 — Supplementary Information 1. [file 41598_2020_71267_MOESM1_ESM.pdf]
